# Cognitive Performance in Early-Onset Schizophrenia and Attention-Deficit/Hyperactivity Disorder: A 25-Year Follow-Up Study

**DOI:** 10.3389/fpsyg.2020.606365

**Published:** 2021-01-14

**Authors:** Merete G. Øie, Kjetil Sundet, Elisabeth Haug, Pål Zeiner, Ole Klungsøyr, Bjørn R. Rund

**Affiliations:** ^1^Department of Psychology, University of Oslo, Oslo, Norway; ^2^Department of Research, Innlandet Hospital Trust, Brumunddal, Norway; ^3^Division of Mental Health, Innlandet Hospital Trust, Ottestad, Norway; ^4^Oslo University Hospital, Oslo, Norway; ^5^Faculty of Medicine, University of Oslo, Oslo, Norway; ^6^Vestre Viken Hospital Trust, Drammen, Norway

**Keywords:** early-onset schizophrenia, adolescence, longitudinal, neurocognition, neurodevelopment, ADHD (Attention Deficit Hyperactivity Disorder)

## Abstract

Early-Onset Schizophrenia (EOS) and Attention Deficit-Hyperactivity Disorder (ADHD) are early- onset neurodevelopmental disorders associated with cognitive deficits. The current study represents the first attempt to compare these groups on a comprehensive cognitive test battery in a longitudinal design over 25 years in order to enhance our knowledge of particular patterns resulting from the interaction between normal maturational processes and different illness processes of these disorders. In the baseline study, 19 adolescents with schizophrenia were compared to 20 adolescents with ADHD and 30 healthy controls (HC), all between 12 and 18 years of age. After 13 years (T2) and after 25 years (T3) they were re-evaluated with the cognitive test battery. A cognitive Composite Score was used in a linear mixed model. The EOS group had a significant cognitive stagnation or deterioration from T1 to T2 compared to HC. However, the EOS group had the most positive change from T2 to T3, supporting a stable level of cognitive performance over the 25 year span. The ADHD group improved or had similar development as the HC group from T1 to T2. They continued to improve significantly compared to the HC group from T2 to T3. Individuals in the EOS group performed more impaired on the cognitive composite score compared to the HC group and the ADHD group at all three time points. Results might indicate a neurodevelopmental pathway of EOS with subnormal cognitive development specific in adolescence. In comparison, the ADHD group had a more consistent cognitive maturation supporting a maturational delay hypothesis of ADHD.

## Introduction

Early-Onset Schizophrenia (EOS) and Attention Deficit-Hyperactivity Disorder (ADHD) are two different disorders, which are considered to have dissimilar etiologies, prognoses, and treatment programs. However, the disorders also share some characteristics. Both are viewed as early-onset neurodevelopmental disorders often persisting throughout the life span (Owen et al., 2011). Moreover, deficits in multiple cognitive domains are central features of both disorders and have been related to functional difficulties in social functioning, education or employment, and independent living (Biederman et al., 2006; Keefe and Harvey, 2012; van Lieshout et al., 2013). However, few studies have investigated whether the two groups differ with regard to how cognitive functions develop from adolescence into adult years. A better understanding of the similarities and differences in the maturation of cognitive function in individuals with EOS and ADHD compared to healthy controls (HC) may enhance our knowledge of particular patterns resulting from the interaction between normal maturational processes and different illness processes of these disorders (Barr, 2001).

Some longitudinal studies comparing adults with schizophrenia with HC report a decline in certain cognitive functions over time (Fett et al., 2019; Zanelli et al., 2019). Some other studies suggest that older patients with schizophrenia (e.g., over 65 years old) show worsening cognitive performance (Harvey, 2001; Thompson et al., 2013). However, most research indicates that schizophrenia is a neurodevelopmental disorder with cognitive impairments that stabilize after illness onset or improve following the first episode of psychosis in adult patients (Rund et al., 2016; Van Haren et al., 2019).

Compared to adult-onset schizophrenia, EOS is associated with greater genetic loading, more pronounced developmental and premorbid deviance, and worse clinical course and outcome compared to adult-onset schizophrenia (Frangou, 2013). The few existing long-term, cognitive follow-up studies of EOS patients compared to HC have reported not only relative stability in some cognitive functions but also abnormal developmental trajectories in cognition throughout late adolescence into early adulthood (Frangou, 2013; Juuhl-Langseth et al., 2014). These results stand in contrast to the stability of cognitive functioning reported in the majority of longitudinal cognitive studies in adults with schizophrenia (Rund et al., 2016).

Longitudinal cognitive studies of individuals with ADHD have documented stability or improvement in cognitive performance through adolescence into young adulthood (Biederman et al., 2009, 2012; Oie et al., 2010; van Lieshout et al., 2019). The results from these studies are in accordance with results from neuroanatomical studies suggesting a maturational lag hypothesis of the pathogenesis of ADHD (Shaw et al., 2007, 2012). This hypothesis suggests a partial or full catch-up of cognitive functioning to the level of healthy controls for cognitive functions. However, questions still exist regarding the persistence and course of these deficits over time in ADHD (van Lieshout et al., 2019).

Limitations in earlier longitudinal studies of adolescents with EOS and ADHD include relatively short follow-up intervals, and few studies have included comparison groups, which is important to be able to control for the potential impact of normative age-associated changes in cognitive functioning. Our research group was the first to compare adolescents with EOS or ADHD and HC on cognitive measures (Øie and Rund, 1999), and to follow them up after 13 years (Oie et al., 2010, 2011). The individuals in the EOS group showed a significant decline or arrest in neurocognitive functioning compared with the other two groups.

In the present study, we wanted to expand our 13-year follow up study (T2) of individuals with EOS, ADHD, and HC to 23–25 years follow-up. In the late twenties the cognitive functions are supposed to be fully matured (Goddings et al., 2012). If there is no decline between 13-year (T2) to 23–25 year follow-up (T3) in the EOS group, it would not support a neurodegeneration progress in EOS. However, if the cognitive decline continues between T2 and T3 in EOS, but not in HC or individuals with ADHD, it may indicate a more general degenerative process in EOS. To the best of our knowledge, no other longitudinal studies have investigated the course of cognitive functioning in adolescents with EOS or ADHD compared to HC over a time period as long as 25 years.

The main aim of the present study is to explore the 23–25-year longitudinal course of cognitive outcome in individuals with EOS or ADHD compared to HC. We predict decline or stagnation in the EOS group on a cognitive composite score from T1 to T2, and both stability (neurodevelopmental disorder) and decline (neurodegeneration) are possible trajectories from T2 to T3. We predict stability or improvement in the ADHD group similar to the HC group at all time points.

## Materials and Methods

### Design and Procedure

A thorough description of the demographic information of the participants from the baseline study (T1) and the 13-year follow-up study (T2) can be found in earlier publications (Øie and Rund, 1999; Oie et al., 2010, 2011). The cross-sectional study at T1 (Øie and Rund, 1999) compared groups of 19 EOS, 20 ADHD, and 30 HC on a comprehensive neuropsychological test battery. All were between 12–18 years at T1. At T1 the EOS group consisted of five female and 14 male patients with a mean age of 16.2 years (SD = 1.1). Fifteen were inpatients and four were outpatients who had never been inpatients. Five of the patients were receiving standard neuroleptic medication (perphenazine, *N* = 3; thioridazine, *N* = 1; zuclopenthixol, *N* = 1) at the time of testing. Three of the patients were drug-free during testing and for a period of at least 5 days before testing. The mean defined daily dose of neuroleptic medication was 0.7 (SD = 0.3) [defined daily dose; WHO Collaborating Centre for Drug Statistics Methodology (World Health Organization, 2009)]. At T1 the ADHD group consisted of 20 males whose mean age was 14.1 years (SD = 1.5). The ADHD group was exclusively male reflecting the fact that ADHD was more commonly diagnosed in boys than girls at T1 (Biederman and Faraone, 2004). All of the ADHD sample were outpatients. None of the patients had a history of psychosis. Comorbidities included oppositional defiant disorder (*N* = 9), developmental reading disorder (*N* = 2), and concurrent oppositional defiant disorder and developmental reading disorder (*N* = 3). Twelve of the participants with ADHD received stimulant medication (11 used methylphenidate, and one used dextroamphetamine). One of the subjects with ADHD received a small dose of haloperidol (1 mg/day) due to tics. Medication in the ADHD patient group was discontinued at least 15 h before testing at both T1, T2, and T3. At T1 the HC group consisted of 14 female and 16 male individuals with a mean age of 15.7 years (SD = 1.6). They were significantly older than the ADHD group (*p* < 0.05). The individuals in the HC group were volunteers attending regular schools at T1. They were screened for mental problems using the Child Behavior Checklist (CBCL), and individuals were excluded if they had a higher raw score than 45 (Øie and Rund, 1999). Diagnoses in both clinical groups were based on fulfilling the diagnostic criteria of the Diagnostic and Statistical Manual of Mental Disorder, Third Edition Revised diagnostic criteria by mental health professionals using semistructured clinical interviews and standardized rating scales. In those EOS cases where the diagnosis was uncertain, the diagnosis was re-evaluated 6 months after discharge and 1 year thereafter. All the diagnoses were confirmed. Diagnostic consensus was investigated in a subsample of 13 patients. Two senior psychologists agreed on the specific schizophrenia diagnosis in 12 (92%) of the cases. Disagreements in diagnosis were discussed between the two, to arrive at a consensus diagnosis. Diagnoses of ADHD subtypes were not made at T1, as this disorder was introduced in a later version of DSM. Exclusion criteria at T1 were: substance abuse, head injury with neurological complications, neurological disorder and IQ < 70.

**FIGURE 1:**
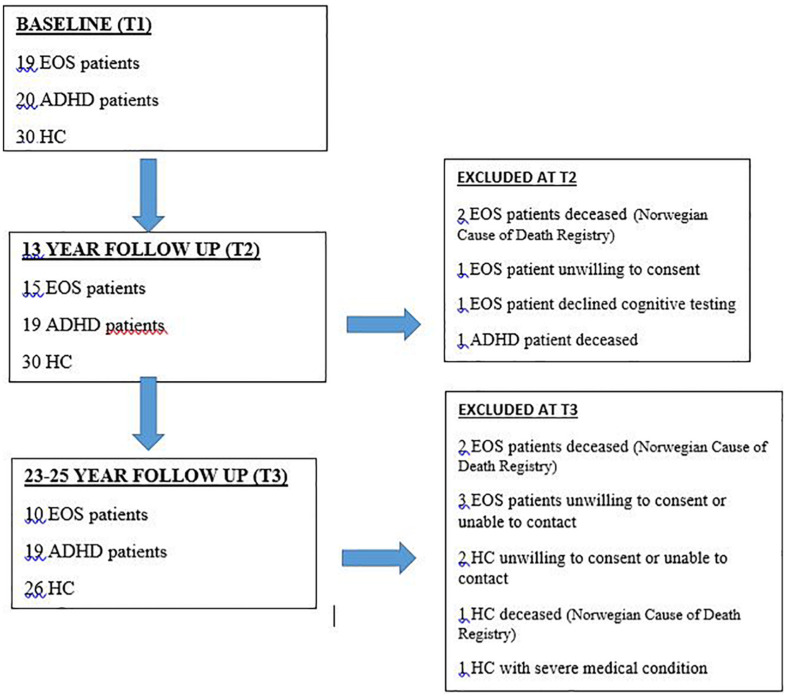
Retention of individuals in the EOS and ADHD groups and HC from baseline to follow-up assessments.

The individuals were reassessed after 13 years (T2), see Øie et al. for details (Oie et al., 2011) and after 23–25 years (T3). At T2, diagnoses in the EOS group were based on the Structured Clinical Interview for the DSM–IV and information from patients’ parents and/or their psychiatrists, nurses, and/or social workers. One psychologist and one psychiatrist reviewed all available information for agreement on the DSM-IV diagnosis. They agreed on the diagnosis in 94% of the cases. Disagreements in diagnosis at T2 were discussed between the two, to arrive at a consensus diagnosis. For a detailed description of the demographic information at T3 see Table 1, and see Table 2 for diagnosis at T1–T3. Figure 1 shows the retention and exclusion of patients groups and HC from baseline through the completion of the third follow-up assessment. Since the time of the first clinical presentation (T1), the EOS patients and the ADHD patients received standard treatment (which did not include cognitive training).

**TABLE 1:** Demographics at T3.

Variable	EOS (*n* = 19)		ADHD (*n* = 19)		HC (*n* = 26)		ANOVA (df = 2,52) F	*p*	Scheffe
Sex (male/female)	6/4		19/0		13/13			0.001	(Fisher)
Hand dominance (R/L)	10/0		16/3		25/1			0.583	(Fisher)

	**Mean**	**SD**	**Mean**	**SD**	**Mean**	**SD**			

Age (y)	38.4	1.1	36.5	1.6	37.9	1.6	6.4	0.003	A < S,HC
Education (y)	10.8	1.5	12.4	2.5	15.7	1.4	29.3	<0.001	S,A < HC
Mother’s education (y)^*a*^	13.3	1.7	12.6	2.5	14.7	2.5	4.0	0.016	A < HC
FSIQ (WASI)^*b*^	94.0	20.5	110.1	10.5	115.1	8.3	10.7	<0.001	S<A,HC
**GAS^*c*^**									
Symptom	55.66	18.3	70.3	11.8	81.0	8.0	17.6	<0.001	S < A,HC
Function	54.9	18.8	71.5	13.6	83.8	6.2	21.7	<0.001	S<A < HC
BPRS^*d*^									
Positive	10.6	5.4							
Negative	5.7	2.6							
Total	40.4	11.9							
ASRS^*e*^			27.8	13.7					
**Medication**									
DDD^*f*^	2.4	2.15	1.8	0.7					
Typical antipsychotic	*n* = 1		–						
Atypical –‘’ –	*n* = 4		*n* = 1						
Both –‘’ –	*n* = 2		–						
Stimulants	–		*n* = 3						
Antidepressant	*n* = 2		*n* = 1						
Benzodiazepine	*n* = 1		*n* = 1						
Mood stabilizer	*n* = 2		–						

*EOS, Early onset schizophrenia; ADHD, Attention Deficit Hyperactivity Disorder; HC, Healthy Controls. ^a^Measured at T2. ^b^Full Scale IQ from the Wechsler Abbreviated Scale of Intelligence, one person in EOS group missing. ^c^Global Assessment Scale. ^d^Brief Psychiatric Rating Scale (Positive Scale = 7 items, Negative Scale = 3 items). ^e^Adult ADHD Self-Report Scale (ASRS). ^f^Defined Daily Doses (WHO Collaborating Centre for Drug Statistics Methodology), EOS: n = 7, ADHD: n = 4.*

**TABLE 2:** Diagnoses at T1, T2, and T3 in the EOS group and the ADHD group.

EOS group	T1	T2	T3
1	schizophrenia disorganized	schizophrenia disorganized	schizophrenia disorganized
2	schizophrenia disorganized	schizophrenia disorganized	schizophrenia disorganized
3	schizophrenia disorganized	schizophrenia disorganized	schizophrenia disorganized
4	schizophrenia paranoid	schizophrenia paranoid	schizophrenia paranoid
5	schizophrenia paranoid	schizophrenia paranoid	schizophrenia paranoid
6	schizophrenia disorganized	schizophrenia disorganized	schizoaffective disorder
7	schizophrenia undifferentiated	schizoaffective disorder	schizoaffective disorder
8	schizophrenia disorganized	schizophrenia disorganized	schizoaffective disorder
9	schizophreniform disorder	recovered	recovered
10	schizophrenia paranoid	recovered	recovered
11	schizophrenia disorganized	schizophrenia disorganized	schizophrenia disorganized unwilling to be tested
12	schizophrenia disorganized	recovered	unwilling to consent or unable to contact
13	schizophrenia disorganized	schizophrenia paranoid	unwilling to consent or unable to contact
14	delusional disorder	unwilling to consent or unable to contact	unwilling to consent or unable to contact
15	schizoaffective disorder	unwilling to consent or unable to contact	unwilling to consent or unable to contact
16	schizophrenia disorganized	schizophrenia disorganized	deceased
17	schizophrenia disorganized	schizophrenia paranoid	deceased
18	schizophrenia paranoid	deceased	deceased
19	schizophrenia disorganized	deceased	deceased

**ADHD group**	**T1**	**T2**	**T3**

1	ADHD	ADHD	ADHD
2	ADHD	ADHD	ADHD
3	ADHD	ADHD	ADHD
4	ADHD	ADHD	ADHD
5	ADHD	ADHD	ADHD
6	ADHD	ADHD	ADHD
7	ADHD	ADHD	ADHD
8	ADHD	ADHD	ADHD
9	ADHD	ADHD	ADHD
10	ADHD	ADHD	ADHD
11	ADHD	ADHD	ADHD
12	ADHD	ADHD	recovered
13	ADHD	ADHD	recovered
14	ADHD	ADHD	recovered
15	ADHD	ADHD	recovered
16	ADHD	recovered	recovered
17	ADHD	recovered	recovered
18	ADHD	recovered	recovered
19	ADHD	recovered	recovered
20	ADHD	deceased	deceased

The T1, T2, and T3 studies were approved by the Regional Committee for Medical Research Ethics in Eastern Norway (REK Øst-Norge REK 1 # 98-05-04,113; 2015/180/REK sør-øst C). The studies were conducted in accordance with the Helsinki Declaration of the World Medical Association Assembly. All subjects were provided written informed consent after receiving a complete description of the study.

### Cognitive Assessments

All individuals were retested at T3 with the same comprehensive neuropsychological test battery as used at T1 and T2. A detailed description of the tests and the procedure is given in Oie et al. (2010, 2011). To reduce the number of statistical comparisons and avoid redundancy, selected test outcome measures were combined into nine cognitive domains according to their putative content, combining the test scores which reflected the same functional domain as described in Oie et al. (2011). Z scores were computed for all tests using the HC group’s raw scores’ means and standard deviations at T1. In cases where higher scores indicated dysfunction, their values were inverted to assure that high scores on the composite scores always indicated better function. The nine cognitive domains consisted of the following measures:

(1) Executive function: Wisconsin Card Sorting Test: Perseverative responses (Heaton, 1981).
(2) Visual memory: Kimura Recurring Figure test: Total correct score (Kimura, 1963).
(3) Verbal memory: California Verbal Learning Test, Total correct words at trial A1-5 (Delis et al., 1987).
(4) Visuomotor processing: The mean of Trail Making Test A, Trail Making Test B, measured as seconds to complete (Reitan and Wolfson, 2004), and Digit Symbol–Coding from WISC–R (Wechsler, 1974) or from WAIS-III (Wechsler, 2003) measured by number of symbols correctly coded in 120 s;
(5) Motor coordination: Grooved Pegboard Test: Mean time in seconds to complete for dominant and non-dominant hand (Reitan and Wolfson, 2004).
(6) Auditory attention: Seashore Rhythm Test: Mean number of correct answers (Reitan and Wolfson, 1993), Digit Span’s maximum span forward and span backward from WISC–R (Wechsler, 1974) or WAIS-III (Wechsler, 2003), and Digit Repetition Test’s proportion of correctly repeated digits with and without distracter digits read in between targets (Oltmanns and Neale, 1975).
(7) Selective attention: Dichotic Listening task: Mean number of correct right ear answers from the Forced Right condition, and number of correct left ear answers from the Forced Left condition (Hugdahl and Andersson, 1986).
(8) Visual attention: Backward Masking task: Mean number of correctly identified digits at the 33 ms and the 49-ms interstimulus intervals (Rund et al., 1996).
(9) Estimated IQ: The WISC–R (T1) and the Wechsler Abbreviated Scale of Intelligence (T2 and T3) subtests Similarities and Block design were used to calculate estimated full-scale IQ (Wechsler, 2007).

The individual cognitive domains were embraced in a composite score because research indicates that the largest amount of variance in cognition deficits in schizophrenia appears to be explained by a global cognitive measure (Rund et al., 2016). The cognitive composite score was calculated as the average of the nine cognitive domains.

### Data Analysis

Demographic and clinical characteristics of the baseline groups were compared by the Fisher exact probability test (nominal variables) and analysis of variance (ANOVA) (continuous variables), the latter followed-up by Scheffe’s *post hoc* tests for group comparisons when adequate. Linear Mixed Models (LMM) was used for longitudinal analysis of individual time course, and to relate change over time to different covariates, in particular group affiliation, HC, EOS, and ADHD. Estimation was based on maximum likelihood (ml) and restricted maximum likelihood (reml), with piecewise linear splines, with one knot at T2 (13 years). Separate random intercepts and slopes were fitted in the first (baseline – 13 years) and second (13–25 years) period, respectively. Parameters of main interest were the fixed effect interaction terms time × group, prior to and following the knot, contrasting the changes in the groups over time. Separate analyses were done with the HC- and the EOS group as reference, to assess all three group-comparisons. The Loss to follow-up was small (see section “Results”), and the usual missing at random assumption (MAR) was thought to be reasonable (the “intention-to-treat” analysis was compared with complete-case). Assessment of fit was done by residuals and outlier checks. Analyses were conducted using the statistical package SPSS for Windows, version 25 (SPSS, Inc., Chicago, IL, United States).

## Results

**FIGURE 2:**
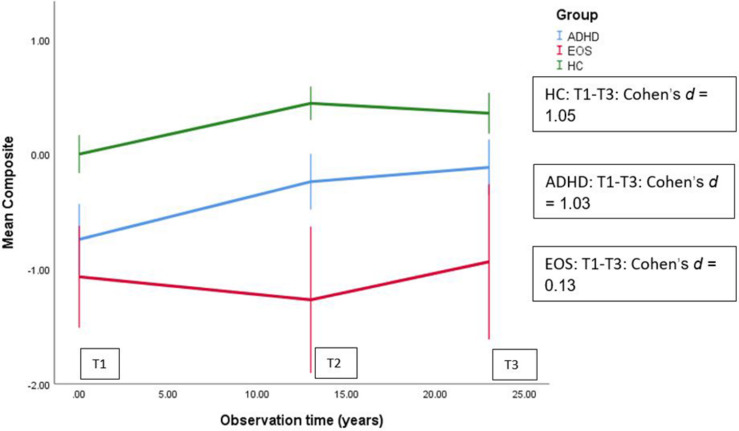
Linear Mixed Models (LMM) used for longitudinal analysis of mean Cognitive Composite score over 25 years in groups of HC (*n* = 30), EOS (*n* = 19) and ADHD (*n* = 20).

In the first period (baseline – 13 years), both the HC and ADHD groups improved (positive slope, main effect) while the EOS group decreased (Table 3). Compared to the HC group, the EOS group had a significantly worse change, with −0.053 units of the Composite score on average per year (*p* < 0.001, 95% CI: −0.079, −0.028) (Table 3 and Figure 2). The EOS group also had a significantly worse change than the ADHD group, with a difference of 0.053 units of the Composite score on average per year in favor of the ADHD group (*p* < 0.001, 95% CI: 0.026, 0.08) – EOS as reference (data not shown). In the second period, however, the EOS group had the most positive change, with the HC group slightly decreasing over time, while the patient groups both had an increase. Both the patient groups had a significant better change than the HC group, with a difference of 0.02 units of the Composite score on average per year for the ADHD group (*p* < 0.05, 95% CI: 0.003, 0.04) and 0.03 units on average per year for the EOS group (*p* < 0.01, 95% CI: 0.01, 0.05) (Table 3). The EOS group also had a more positive change than the ADHD group, but not significant (data not shown). The effect size estimate (η^2^ = 0.11) for the Composite score indicates a major different trajectory between groups. For the EOS group, the change from T1 to T3 was not significant (Cohen’s *d* = 0.13), but for the HC group and the ADHD group, there was a significant and large improvement from T1 to T3 (HC; Cohen’s *d* = 1.05, and ADHD; Cohen’s *d* = 1.03).

**TABLE 3:** Fixed effects in a Linear Mixed Model (LMM) with outcomes of Cognitive Composite score, with follow-up over 23–25 years in groups of HC (*n* = 30), EOS (*n* = 19), and ADHD (*n* = 20).

Cognitive Composite score			
	Estimate^†^	SE	95% CI
**Main effect group**			
ADHD	−0.68**	ADHD	−0.68**
EOS	−1.02***	EOS	−1.02***
HC	0 (Ref)		
**Main effect time**			
Time ≤ 13 years	0.034***	0.007	0.019, 0.049
Time > 13 years	–0.008	0.005	−0.019, 0.003
**Interaction, group × time ≤ 13 years**			
ADHD	–0.0004	0.011	−0.024, 0.023
EOS	−0.05***	0.013	−0.079, −0.028
HC	0 (Ref)		
**Interaction, group × time > 13 years**			
ADHD	0.02*	0.008	0.003, 0.04
EOS	0.03**	0.01	0.01, 0.05
HC	0 (Ref)		

†: adjusted for education at baseline, *p ≤ 0.05, **p ≤ 0.01, ***p ≤ 0.001.

See Tables 4, 5 for results on the individual cognitive tests and cognitive domains, and differences between groups over time for those individuals that participated on all the test points (i.e., without the individuals that died or declined to be retested).

**TABLE 4:** Cognitive test scores at T1, T2, and T3 for individuals in the EOS, ADHD, and HC groups participating at all three test times.

	EOS *N* = 10						ADHD *N* = 19						HC *N* = 26						Group df = 2,52		Time df = 2,51		Time x Group df = 4,102		

Domains	T1 Mean	SD	T2 Mean	SD	T3 Mean	SD	T1 Mean	SD	T2 Mean	SD	T3 Mean	SD	T1 Mean	SD	T2 Mean	SD	T3 Mean	SD	F	p	F	p	F	p	η^2^
**Executive function**																									
*WCST*																									
Perseverative r	20.9	11.4	22.6	15.3	16.7	10.2	19.0	8.4	12.4	5.0	8.7	4.9	15.9	6.5	9.7	4.9	6.7	3.9	9.8	0.001	19.6	0.001	1.4	0.238	0.06
**Visual memory**																									
*Kimura*																									
Recognition	25.7	11.3	26.6	10.5	28.4	6.4	34.7	10.3	31.7	9.1	33.0	8.9	38.9	6.4	37.2	6.3	38.4	7.6	10.5	0.001	0.9	0.415	0.3	0.856	0.01
**Verbal memory**																									
*CVLT*																									
Total 1–5	54.8	10.5	46.9	11.3	48.5	9.6	50.4	9.0	51.6	6.9	51.7	9.4	59.9	8.0	61.5	8.9	55.5	8.9	9.0	0.001	2.3	0.107	4.2	0.004	0.14
**Visuomotor Processing**																									
*TMT A*	30.1	11.1	30.8	11.4	33.6	9.0	27.0	5.2	26.8	7.7	22.5	7.6	23.6	6.2	20.7	5.3	21.7	5.0	11.6	0.001	0.3	0.733	3.1	0.017	0.11
*TMT B*	77.3	20.3	74.4	42.7	90.2	51.6	80.0	31.9	62.1	21.7	56.3	19.9	60.7	20.4	45.6	13.9	55.4	22.4	5.8	0.006	8.4	0.001	2.8	0.029	0.10
*Digit Symbol Correct*	71.2	16.9	85.2	12.1	86.7	12.8	64.8	17.2	65.5	16.0	63.9	17.3	88.05	17.4	85.2	12.1	86.7	12.8	20.0	0.001	6.3	0.004	2.5	0.049	0.09
**Motor Coordination**																									
*Grooved Peg*																									
Dominant	71.3	9.6	73.3	27.3	73.0	18.5	66.6	11.6	65.6	11.3	62.1	10.4	59.7	8.4	54.7	7.5	54.0	5.3	11.4	0.001	2.6	0.085	1.2	0.290	0.05
Non-dominant	89.3	19.1	94.6	58.6	84.9	28.8	78.2	14.4	74.4	18.9	70.6	18.5	69.5	8.4	63.8	8.3	62.0	9.4	7.7	0.001	7.2	0.002	0.41	0.798	0.02
**Auditory Attention**																									
*Seashore*																									
Correct	24.8	2.3	23.2	5.9	24.8	4.2	25.3	3.0	26.0	2.1	25.5	2.9	27.0	3.1	27.2	2.9	27.3	2.5	4.2	0.020	0.8	0.469	2.1	0.090	0.08
*Digit Span*																									
Forward max	5.7	1,0	5.6	1.1	6.2	1.0	5.8	1.2	6.1	1.4	6.1	1.3	6.3	1.3	6.5	1.2	6.7	1.3	2.1	0.131	2.8	0.069	0.6	0.624	0.03
Backward max	4.2	2.0	4.2	1.3	4.6	1.5	3.9	1.0	4.3	1.1	4.6	1.3	4.7	1.4	4.5	1.3	4.8	0.9	1.1	0.350	2.4	0.102	0.6	0.649	0.025
*Digit Repetition*																									
Without dist	74.8	22.7	81.0	13.9	80.9	16.3	66.6	20.0	83.0	8.9	81.3	13.9	86.7	10.9	88.7	8.2	89.7	8.2	3.9	0.026	0.2	0.001	1.9	0.122	0.07
With dist	71.7	23.4	83.4	15.7	81.0	18.6	63.3	22.0	83.9	13.6	75.7	18.6	83.2	14.7	93.7	7.7	89.6	10.0	6.3	0.004	0.1	0.001	3.2	0.017	0.11
**Selective Attention**																									
*Dichotic List*																									
FR, REA	13.1	4.2	13.2	2.0	15.3	6.1	16.2	4.6	19.1	4.9	19.6	3.7	15.1	4.3	20.3	4.3	19.6	4.1	7.2	0.002	10.6	0.001	2.3	0.065	0.082
FL,LEA	14.3	14.3	12.6	4.9	12.6	6.8	14.0	4.8	13.8	6.0	14.3	4.6	13.7	3.8	17.5	4.9	14.9	5.5	1.6	0.205	0.6	0.574	2.2	0.076	0.079
**Visual Attention**																									
*Backward masking*																									
33 ms	5.5	2.6	6.9	5.9	4.2	2.4	3.9	3.1	9.1	5.3	5.5	4.5	5.5	4.1	7.7	3.3	6.5	3.6	0.3	0.720	10.3	0.001	2.4	0.053	0.09
49 ms	7.4	3.7	8.8	5.0	6.0	2.5	7.2	5.3	10.9	5.0	7.6	3.9	10.2	5.0	9.2	5.1	9.0	4.7	1.0	0.371	3.3	0.044	2.5	0.045	0.09

*Executive function: Wisconsin Card Sorting Test: Perseverative responses; Visual memory: Kimura Recurring Figure test: Total correct score; Verbal memory: California Verbal Learning Test, Total correct words at trial A1-5; Visuomotor processing: The mean of Trail Making Test A, Trail Making Test B, measured as seconds to, and Digit Symbol–Coding from WAIS-III measured by number of symbols correctly coded in 120 s; Motor coordination: Grooved Pegboard Test: Mean time in seconds to complete for dominant and non-dominant hand; Auditory attention: Seashore Rhythm Test: Mean number of correct answers, Digit Span’s maximum span forward and span backward from WAIS-III, and Digit Repetition Test’s proportion of correctly repeated digits with and without distracter digits read in between targets; Selective attention: Dichotic Listening task: Mean number of correct right ear answers from the Forced Right condition, and number of correct left ear answers from the Forced Left condition; Visual attention: Backward Masking task: Mean number of correctly identified digits at the 33 ms and the 49-ms interstimulus intervals. Due to missing data on some tests, the number of individuals in each within each group will vary slightly on some domains/Composite score.*

**TABLE 5:** Cognitive Domain Scores and Composite score at T1, T2, and T3 for individuals in the EOS, ADHD and the HC groups participating all three test times.

	EOS N = 10						ADHD N = 19						HC N = 26						Group df = 2,50		Time df = 2,49		Time x Group df = 2,49		

Cognitive domains	T1 Mean	SD	T2 Mean	SD	T3 Mean	SD	T1 Mean	SD	T2 Mean	SD	T3 Mean	SD	T1 Mean	SD	T2 Mean	SD	T3 Mean	SD	*F*	*p*	*F*	*p*	*F*	*p*	*η^2^*
Executive function	–0.90	1.7	−0.92	2.1	−0.21	1.6	−0.49	1.1	0.52	0.68	1.1	0.69	−0.05	0.99	0.97	0.69	1.39	0.55	10.78	0.000	24.49	0.505	1.25	0.294	0.05
Visual memory	–0.2.08	1.7	−1.94	1.6	−1.65	1.0	−0.67	1.6	−1.13	1.4	−0.93	1.4	−0.01	1.0	−0.28	0.98	−0.09	1.18	10.48	0.000	0.98	0.036	0.33	0.856	0.01
Verbal memory	–0.66	1.3	−1.63	1.4	−1.43	1.2	−1.20	1.1	−1.05	0.85	−1.04	1.2	−0.04	0.98	0.15	1.1	−0.57	1.1	8.94	0.000	2.33	0.107	4.15	0.004	0.14
Visuomotor processing	–0.97	0.94	−0.91	1.5	−1.22	1.6	−0.97	1.0	−0.19	1.0	0.23	1.0	0.00	0.80	0.97	0.71	0.80	0.83	14.16	0.000	9.8	0.000	5.19	0.001	0.18
Motor coordination	–1.6	1.4	−2.03	4.7	−1.47	2.5	−0.76	1.3	−0.49	1.6	−0.10	1.5	0.09	0.77	0.67	0.79	0.81	0.74	9.68	0.000	6.98	0.002	0.49	0.745	0.01
Auditory Attention	0.77	1.0	−0.60	1.1	−0.37	1.0	−1.0	1.0	−0.25	0.65	−0.40	0.95	−0.05	0.73	0.18	0.63	0.20	0.58	5.65	0.006	10.29	0.001	2.44	0.052	0.09
Selective Attention	–0.23	0.75	−0.42	0.50	−0.16	0.63	0.12	0.98	0.47	1.05	0.59	0.78	−0.05	0.89	1.07	1.02	0.66	1.08	4.65	0.014	3.73	0.031	3.45	0.011	0.12
Visual Attention	–0.29	0.63	0.01	1.2	−0.59	0.45	−0.52	0.84	0.49	1.09	−0.27	0.90	−0.03	0.93	0.14	0.84	−0.01	0.85	0.66	0.523	7.53	0.001	3.07	0.020	0.11
Estimated IQ	–1.1	1.2	−1.29	1.2	1.26	1.4	−0.62	0.82	−0.50	0.55	−0.20	0.68	0.01	1.0	0.08	0.56	0.13	0.52	10.15	0.000	2.72	0.076	1.89	0.118	0.07
Composite Score	–1.0	0.76	−1.02	1.3	−0.88	1.1	−0.71	0.61	−0.25	0.51	−0.13	0.51	−0.01	0.42	0.48	0.37	0.40	0.36	18.3	0.000	14.5	0.000	2.75	0.033	0.11

*Due to missing data on some tests, the number of individuals in each within each group will vary slightly on some domains/Composite score.*

## Discussion

As predicted, the EOS group had a significant stagnation or deterioration on the composite score in the first period from T1 to T2 compared to HC. However, in contrast to our expectation, the EOS group had the most positive change in the second period (from T2 to T3), with the HC group slightly decreasing over time. The results do not support a neurodegenerative model of EOS but suggest a premature arrest, or slowing, of normal cognitive development occurring mainly in their twenties, but no decline after that. Thus, our results support the neurodevelopmental model of EOS (Rund, 2018). As expected, the individuals in the EOS group performed more impaired on the cognitive composite score compared to the HC group and the ADHD group at all three time points.

The cognitive maturation in the ADHD group was not significantly different from the HC group from T1 to T2, but they continued to improve on the composite score compared to the HC group from T2 to T3. Thus, we found that cognition continues to mature in the ADHD group after the mid-20 s which is considered the “peak” of executive functions development (De Luca and Leventer, 2010). Our results support a model of ADHD that indicate a cognitive developmental lag that reduces with age. In a separate study on the same individuals, we found a selective decline in performance from T2 to T3 for the ADHD group compared to the HC group on a working memory test (Torgalsbøen et al., 2019). Thus, the individuals in the ADHD group continued to display working memory deficits, also in adulthood.

The EOS group had a significant worse cognitive change compared to the ADHD group in the first period, while in the second period both the patient groups had a significantly better change compared to HC. The cognitive results support the notion that both EOS and ADHD are neurodevelopmental disorders, but that the EOS group stagnates in their cognitive development for a period from adolescence to young adulthood (T1 to T2), while the ADHD group has a more consistent cognitive maturation up to our last measure time point at T3. Further, the ADHD group seems to catch up with the HC group in their thirties (T3) regarding most cognitive functions, but the EOS group does not. Thus, our data support a maturational delay hypothesis of the pathogenesis of ADHD (Shaw et al., 2007) compared to a deviation from normal cognitive development in the twenties in EOS (Oie et al., 2010).

What can explain why the EOS group did not have the same cognitive trajectory as the HC and the ADHD group in the first period, but a more positive development in cognition in the second period? The individuals in the EOS group became ill at a young age. Early onset of the illness and cognitive difficulties may halt their development in social and academic areas. Brain functions mature extensively during adolescence to early adulthood through continuous interactions with the environment (Casey et al., 2008; Sakurai et al., 2015). The individuals with EOS become seriously ill in this important maturation period, and at the same time, they also have to cope with psychotic symptoms and having a serious illness. This may have led to high levels of stress interacting with the disease process leading to disrupted normal development of brain functions. We have earlier reported that the individuals with EOS at T1 had considerably higher levels of internalizing problems including depressive symptoms compared to the HC group and the ADHD group (Oie et al., 2011). When depression is investigated longitudinally in schizophrenia, up to 80% of patients experience a clinically significant depressive episode at one or more time points during the early phase (Upthegrove et al., 2017). Depression may negatively affect cognition (Douglas and Porter, 2009). A longitudinal study on depressive symptoms in adults with first episode of schizophrenia has reported that depressive symptoms decreased during a 10 year follow-up period (Sönmez et al., 2016). Thus, both stress and depression in the EOS group during the first period may have negatively affected the cognitive functions more than in the second period. It may be that the cognitive functions are more vulnerable to negative environmental and/or illness factors in the time period from T1 to T2 and that the cognitive development is interrupted. After many years with illness (T3), the EOS group may have learned how to live better with their illness, experiencing less depression and stress and to have more capacity to efficiently use their cognitive resources.

In contrast, it is reasonable to believe that adolescents with ADHD are more often at school and with friends, and are more exposed to various stimuli than individuals with EOS. Several of the patients in the current EOS group moved away from home to stay in institutions, while in the ADHD group they could all continue to live at home and in familiar surroundings. Schizophrenia is regarded as a more serious illness than ADHD, and there is also more knowledge in the population about ADHD because it is a more common disorder. Thus, the ADHD group may have experienced less stress and less comorbid depression, and less interruption with the cognitive maturation, in the first period compared to the EOS group. It is also possible that adolescents with EOS receive less help and facilitation for cognitive difficulties compared to adolescents with ADHD.

Strengths of the study include a long follow-up time (23–25 years), a relatively high retention rate (19/20 ADHD individuals, 26/30 HC), and inclusion of the same HC group at the three time points. The inclusion of HC makes it possible to determine whether the trajectory found in the patient groups was different from the normal cognitive maturation. The cognitive test battery constituted a comprehensive cognitive assessment, and the same test battery was administered at all three time points. Further, the long intervals between assessments may minimize practice effects. The drop-out of some of the individuals was to some extent accounted for in the LMM under the MAR assumption.

The small patient sample sizes limit the generalizability of our results and reduce the statistical power to detect changes in cognitive performance. The small sample size is due to the lower incidence and prevalence of EOS. The ADHD group consisted of only males. Further, another limitation is that there was a significant difference in age distribution between the ADHD and HC groups. In the analyses, we did not control for the use of medication, and this could possibly have affected the cognitive results. However, a meta-analysis of randomized clinical trials of second-generation antipsychotic effects on cognition in patients with schizophrenia did not show any drug having a uniform positive cognitive profile (Nielsen et al., 2015). Further, changes in symptoms may possibly have an impact on the changes in cognition. We decided to include all available individuals from the EOS and the ADHD groups regardless of whether they had recovered and did not meet the diagnostic criteria for schizophrenia or ADHD at follow-up (T3). We also included the recovered individuals because this was in accordance with what was done in the 13-year longitudinal follow-up and because the primary objective of the study was to investigate how cognition in adolescents with EOS or ADHD developed over time regardless of diagnostic status at follow-up. Several studies have shown that it is possible for patients with schizophrenia to recover (Hegelstad et al., 2012; Jääskeläinen et al., 2013; Lally et al., 2017; Torgalsbøen et al., 2018). The percentage of those who recover varies from 15 to 55 percent depending on the criteria used for recovery. Thus, our three clinical recovered cases out of 19 are in line with other research. Furthermore, analyzes with ANOVA showed that there were no significant mean differences between the recovered and the non-recovered individuals on the Composite scores at T1, T2, or T3. Another possible limitation might be that the individuals in the EOS group who either died or declined to be retested could be the more severe cases. However, we have no information indicating that this was the case. Due to data protection privacy concerns, we could not describe these patients in further detail. On the other hand, it is also possible that they declined because they are doing well and do not want to be reminded of their previous illness. Thus, it is difficult to establish the reasons why individuals decline to participate in follow-up studies. Also, as shown in Table 5, the average of the Composite score at T1, T2, and T3 in the EOS group without the individuals who died or declined to be retested, are quite similar to those shown in Figure 2. There are some disadvantages to using Composite scores as they may mask important differences apparent in the individual cognitive domains, which may have changed in different directions. It is also possible that non-cognitive factors such as anxiety and effort in the test situation may have affected the test results.

Using WCST Perseverative responses as the sole measure of executive functioning may also be regarded as a limitation. WCST lacks cognitive specificity as performance has been associated with deficits in set-shifting, working memory, and general cognitive ability (Donohoe et al., 2005). As such, the scores presented do not fully represent a composite index of “executive functioning,” but only one facet of executive functioning. In addition, the significantly lower T2 and T3 scores for WCST Perseverative responses for the ADHD group and the HC group may overestimate “executive functioning” performance because they might develop test strategies and remember the test items better than people with schizophrenia and therefore perform better in the second and third assessment (Chiu and Lee, 2019). As such, the differences between the EOS group versus the ADHD and HC groups for T2 and T3 WCST performance may not reflect changes in Executive Functioning in any of the groups.

In conclusion, our results might indicate a neurodevelopmental pathway of EOS with subnormal cognitive development specific in adolescence. In comparison, the ADHD group had a more consistent cognitive maturation supporting a maturational delay hypothesis of ADHD. Our results may underline the importance of treatment strategies to alleviate the subnormal development of cognitive functions and improve the relatively stable cognitive deficits in the early illness phase of EOS. However, our results must be interpreted with caution due to small patient sample sizes and other possible limitations.

## Data Availability Statement

The datasets presented in this article are not readily available because we have not received permission to share data with others, in which case it must be applied for again to the ethics committee. Requests to access the datasets should be directed to MØ, m.g.oie@psykologi.uio.no.

## Ethics Statement

The T1, T2, and T3 studies were approved by the Regional Committees for Medical Research Ethics in Eastern Norway (REK Øst-Norge REK 1 # 98-05-04,113; 2015/180/REK sør-øst C). The patients/participants provided their written informed consent to participate in this study.

## Author Contributions

BR planned the baseline study, while MØ planned the follow-up studies. MØ undertook the literature search, collected the data in the baseline and the follow-up studies and wrote the first draft of the manuscript. MØ and OK conducted the statistical analyses. KS assisted with statistical analysis. All authors made contributions to interpretations and content, and approved the final manuscript.

## Conflict of Interest

The authors declare that the research was conducted in the absence of any commercial or financial relationships that could be construed as a potential conflict of interest.
